# Distributed SLAM Using Improved Particle Filter for Mobile Robot Localization

**DOI:** 10.1155/2014/239531

**Published:** 2014-04-27

**Authors:** Fujun Pei, Mei Wu, Simin Zhang

**Affiliations:** School of Electronic Information & Control Engineering, Beijing University of Technology, Beijing 100124, China

## Abstract

The distributed SLAM system has a similar estimation performance and requires only one-fifth of the computation time compared with centralized particle filter. However, particle impoverishment is inevitably because of the random particles prediction and resampling applied in generic particle filter, especially in SLAM problem that involves a large number of dimensions. In this paper, particle filter use in distributed SLAM was improved in two aspects. First, we improved the important function of the local filters in particle filter. The adaptive values were used to replace a set of constants in the computational process of importance function, which improved the robustness of the particle filter. Second, an information fusion method was proposed by mixing the innovation method and the number of effective particles method, which combined the advantages of these two methods. And this paper extends the previously known convergence results for particle filter to prove that improved particle filter converges to the optimal filter in mean square as the number of particles goes to infinity. The experiment results show that the proposed algorithm improved the virtue of the DPF-SLAM system in isolate faults and enabled the system to have a better tolerance and robustness.

## 1. Introduction

Autonomous localization is a process of determining the robot's position without the use of any a priori information external to the robot except for what the robot senses about its unknown environment. This is also known as Simultaneous Localization and Mapping (SLAM), as introduced in [1, 2], where the robot has a capability for online map building, while simultaneously using the generated map to estimate and correct errors in the navigation solution obtained. It is well known that SLAM using an optimal filter, such as EKF-SLAM [3, 4], PF-SLAM [5], and FastSLAM [6–8], has been applied in many systems and solved a lot of pragmatic problems.

However, SLAM based on single centralized filter must reconfigure the entire system equation when the feature points change, which cause an exponential growth in computation quantities and difficulties in isolate potential faults. To tackle these limitations of centralized filter, Won et al. proposed a distributed particle filter for implementing vision-SLAM [9, 10]. Different from centralized particle filter, the distribute SLAM divides particle filter to some “feature point blocks” and some “landmark blocks,” which make it easier to design the filter and simplify the calculation. The simulation results showed that the distributed SLAM system has a similar estimation performance and requires only one-fifth of the computation time compared with centralized particle filter.

However, particle impoverishment is inevitably induced because of the random particles prediction and resampling applied in generic particle filter [11], especially in SLAM problem that involves a large number of dimensions. If the generated particles are too far from the likelihood distribution, the particle weights will approach zero with only a few particles carrying significant weights, making other particles not efficient to produce accurate estimation results. In general, there are two types of improved methods for these problems.

1. There are many improved resampling methods that have been discussed, such as binary search [11], systematic resampling [12], and residual resampling [13]. However, these methods are not ideal because the copied samples are no longer statistically independent after resampling sample. Therefore the convergence result obtained from previous step will be lost, which is called losing sampling diversity. These problems caused the same problem in distributed particle filter used for SLAM problem.
2. Another improved method was optimizing the proposal distribution using optimal estimation algorithms such as extended Kalman [14], or unscented Kalman filter [15, 16]. Since these methods, especially for unscented PF, estimate the proposal distribution in order to minimize the variance of the importance weights, which ensure that the weights are upper bounded. Thus it is not a surprise that unscented particle filter yields better proposal distribution than other methods.

In this paper, the improved particle filter was proposed to estimate the state vector of distributed SLAM. The performance of the particle filter is affected by several factors in its operation. First, the important function used to calculate the probability of the particles in local filters. To calculate the probability of these particles, the variance of these particles has to be estimated. The precision of the estimation results will be improved if the estimation variance is close to the real variance of the particles. By changing the parameter from a set of constants to a variable, determined by the variance of the particles in each local filter. Second, the fusion algorithm is applied to calculate the estimation results in the master filter. In distributed filter, the estimation results of each local filter are transmitted to the master filter to calculating the estimation results by fusion algorithm. There are two methods for calculate the weights of the local filters: the precision of the local filter and the number of effective particles. Both of these methods have their advantages and drawbacks. Then an improved method which mixed these two methods was proposed to calculate the weights of the local filters. In order to test the accuracy and tolerance of the proposed algorithm, two experiments were carried out. Experiment results show that the improved particle filter has better performance in accuracy and tolerance.

The remainder of this paper is organized as follows.  Section 2 explains the SLAM system based on laser sensor and Section 3 introduced the DPF applied in SLAM system. Section 3 proposes an improved DPF-SLAM system. Section 3 introduced structure of the improved DPF-SLAM system.  Section 4 presents the results of simulation and Section 5 states our conclusion.

## 2. Distributed SLAM System

### 2.1. The Centralized SLAM System

**Figure 1:**
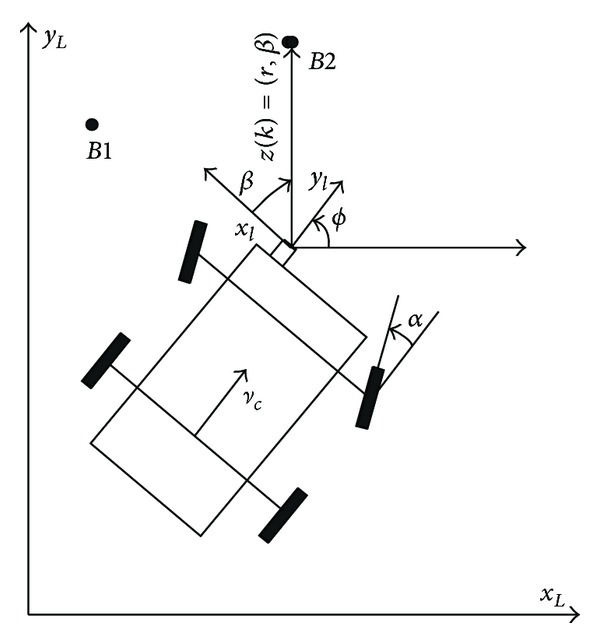
Vehicle system.

In SLAM system, although the absolute position of the autonomous robot is not accessible, it is possible to use the information of indirect observation to estimate the position of the autonomous robot and maintain the error in a small range. SLAM, however, is rarely used alone but is combined mostly with dead reckoning (DR) or inertial navigation system (INS), because it has a low update rate for providing navigation information. The composing of SLAM system is dead reckoning (DR) and laser system, which is shown in Figure 1.

A SLAM system model combines the robot's kinematics model with the feature point model. The state vectors of the kinematics model represent position and azimuth; those of the feature point model refer to the feature point position, which is assumed to be a fixed point and its error is therefore modeled into a random constant. Equation (1) describes a system model used in SLAM system as follows:

(1) $$\begin{array}{c} \dot{x}=Fx+GW, \end{array}$$

where

(2) $$\begin{array}{c} x={\left[\begin{array}{ccccc} x_{r} & m_{1} & m_{2} & \cdots & m_{n} \end{array}\right]}^{T},\\ {\dot{x}}_{r}=\left[\begin{array}{c} {\dot{x}}_{r}\\ {\dot{y}}_{r}\\ {\dot{\phi }}_{r} \end{array}\right]=f\left(x\right)+\gamma =\left[\begin{array}{c} v_{c}\cdot \cos\left(\phi \right)\\ v_{c}\cdot \sin\left(\phi \right)\\ \frac{v_{c}}{L}\cdot \tan\left(\alpha \right) \end{array}\right]+\gamma . \end{array}$$

${\dot{x}}_{r}$, ${\dot{y}}_{r}$, ${\dot{z}}_{r}$ is the change of the state. *L* is the distance between wheels. *γ* is the of the noise in DR system. **m**_1:*n*_ is feature point position.

The ranging and bearing of the feature points are utilized to configure a SLAM system, and these values are determined by the azimuth of the robot and the relative position between the robot and feature points. The ranging and bearing noises can be assumed to be in a simple relationship of summation so that the observation equation is converted into following nonlinear equation:

(3) $$\begin{array}{c} z\left(k\right)=h\left(x_{r}\left(k\right),m_{i}\left(k\right)\right)+v\left(k\right), \end{array}$$

where **z**, **x**_*r*_, and **m**_*i*_ stand for the observed value, the vehicle related state, and the position of the *i*th feature point, respectively. The measurement noise, described as **v** *N*(0, *R*), can be assumed to be normal distribution with the covariance of *R*.

The observed value **z**(*k*) = (*r*, *β*) received by the laser sensor is the ranging and bearing information of different feature points *m*_*i*_ (*i* = 1 … *n*), where *r* is the distance between sensor and feature points and *β* is the bearing measured by the laser sensor in robot coordinate. When multiple landmarks were observed, the observation vector will be represented by the following:

(4) $$\begin{array}{c} z=\left[\begin{array}{c} z_{1}\\ z_{2}\\ \vdots \\ z_{n} \end{array}\right]=\left[\begin{array}{c} h\left(x_{r}\left(k\right),m_{1}\right)\\ h\left(x_{r}\left(k\right),m_{2}\right)\\ \vdots \\ h\left(x_{r}\left(k\right),m_{n}\right) \end{array}\right]+v\left(k\right), \end{array}$$

where

(5) $$\begin{array}{c} h\left(x_{r}\left(k\right),m_{i}\right)=\left[\begin{array}{c} \sqrt{{\left(x_{i}-x_{L}\right)}^{2}+{\left(y_{i}-y_{L}\right)}^{2}}\\ a\tan\left(\frac{\left(y_{i}-y_{L}\right)}{\left(x_{i}-x_{L}\right)}\right)-\phi_{L}+\frac{\pi }{2} \end{array}\right]+v\left(k\right). \end{array}$$

(*x*_*i*_, *y*_*i*_) is the coordinate of the *i*th feature point, *i* = 1,2,…, *n* is the number of the feature points measured, and *x*_*L*_, *y*_*L*_, *ϕ*_*L*_ is the location of the sensor in robot coordinate.

### 2.2. The Structure of Distributed SLAM System

Based on the system model in (1), it is evident that is a nonlinear system. The most widely applied solutions of SLAM system are EKF, UKF, and PF. However, the SLAM system based on EKF, UKF, or PF uses only one centralized filter, which uses a single filter to estimate all states of SLAM system. With the running of the robot, the number of feature points which can be observed will change. Therefore, the structure of centralized SLAM system must be reconfigured whenever the observation environment of the landmarks changes. On the other hand, these centralized filters will cause quantum of calculation to extend with the state vector increasing. Furthermore, centralized filter is hard to detect and isolate possible faults.

To tackle the drawbacks of centralized SLAM system, Won et al. proposed SLAM system based on distributed particle filter [10], which replaced the single centralized particle filter with local particle filters and a master filter. This technique avoids the necessity to change the structure of filter with the dimension of observation. And each local particle filter can get its own estimation result separately. Thus, even if some of the observations of the local filter contain large errors, it is easy to eliminate its effect.

Won et al. proposed the structure of distributed SLAM system including feature pint position subsystem and robot's position and attitude subsystem. The distributed SLAM system model of the vehicle's position estimated system is described in the following:

(6) x˙v=f(xv)+γz1=h(xv(k),m1)+v1(k)x˙v=f(xv)+γz2=h(xv(k),m2)+v2(k) ⋮x˙v=f(xv)+γzn=h(xv(k),mn)+vn(k).

The estimation results were feedback to correct the position of vehicle.

The system model of the distributed landmarks position estimation system is described in the following:

(7) m˙1=0z1=h(xv(k),m1)+v1(k)m˙2=0z2=h(xv(k),m2)+v2(k) ⋮m˙2=0z2=h(xv(k),m2)+v2(k).

The estimation results of the feature point position subsystem are used to update the environment map. And the estimation results of the robot's position and attitude subsystem are transmitted into the master filter, which fused estimation results from each of the local filters to estimate robot's position and attitude. When fusing state vectors from the local filters, the master filter applies different weights to each local filter. Here the weights are set based on the innovation of each local filter.

## 3. The Improved Distributed Particle Filter

The distributed SLAM reduces the computation quantities and has similar estimation performance compared with centralized SLAM. However, there are some limitations inherent in particle filter, such as particle impoverishment and losing sampling diversity, which will cause divergence of the filter.

In this section, the improved distributed particle filter was proposed to improve the performance of the distributed particle filter in two different aspects. First, the improved important function method in the local particle filters was proposed by adaptive adjusting of the estimation value of covariance. Second, an information fusion method was proposed by mixing the innovation and the number of effective particles methods.

**Figure 2:**
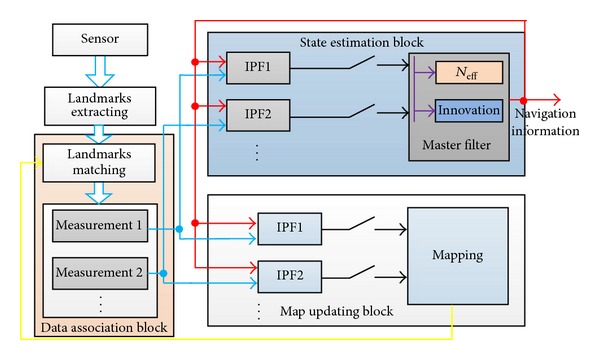
The structure of improved DPF SLAM system.

The structure of improved DPF, proposed in this paper to realize a SLAM system, is described in Figure 2. In Figure 2, there are two subsystem blocks in the IDPF-SLAM system: state subsystem block and mapping subsystem block. State subsystem estimates the state of the robot using the proposed Improved DPF. Mapping subsystem estimates the position of the landmarks and updates the map of the environment using Improved DPF.

### 3.1. The Improved Important Function

Roughly speaking, particle filters are numerical algorithms to approximate the conditional distribution by an empirical distribution, constituted by a cloud of particles at each time instant. Thus one important feature of the particle filter is that a series of particles are generated randomly {*x*_0:*t*_^(*i*)^; *i* = 1,…, *N*} from posterior distribution which is used to map integrals to discrete sums. In other words, the posterior can be approximated by the following empirical estimation:

(8) $$\begin{array}{c} \hat{p}\left(x_{0:t}\;|\;y_{1:t}\right)=\frac{1}{N}\overset{N}{\underset{i=1}{\sum }}\delta_{x_{0:t}^{\left(i\right)}}\left(dx_{0:t}\right), \end{array}$$

where *δ*_*x*_0:*t*_^(*i*)^_(*d* **x**_0:*t*_) denotes the Dirac delta function.

Base on (8), (9) can be estimate by (10)

(9) $$\begin{array}{c} E\left(g_{t}\left(x_{0:t}\right)\right)=\int g_{t}\left(x_{0:t}\right)p\left(x_{0:t}\;|\;y_{1:t}\right)dx_{0:t} \end{array}$$

(10) $$\begin{array}{c} \bar{E\left(g_{t}(x_{0:t})\right)}=\frac{1}{N}\overset{N}{\underset{i=1}{\sum }}g_{t}\left(x_{0:t}^{\left(i\right)}\right). \end{array}$$

The particles **x**_0:*t*_^(*i*)^ are assumed to be independent and identically distributed.

According to the law of large numbers, we have $\bar{E\left(g_{t}(x_{0:t})\right)}\underset{N\to \infty }{\overset{\;\;}{\to }}E\left(g_{t}(x_{0:t})\right)$. And if the posterior variance of **g**_*t*_(**x**_0:*t*_) is bounded which means. Consider

(11) N(E(gt(x0:t))−E(gt(x0:t)))  →  N→∞N(0,varp(x0:ty1:t)(gt(x0:t))).

Thus, if we assumed that the distribution of the particles obeys gauss distribution, whose mean and variance are observation, the probability of the particles can be estimated by (12). This is the most popular used method to generate the probabilities of the particle filters:

(12) $$\begin{array}{c} w_{k}^{i^{*}}=\frac{1}{{\left(2\pi \right)}^{n_{v}/2}\sqrt{\det R_{k}}}e^{-(1/2){\left(z_{k}-h_{k}(x_{k}^{i^{*}})\right)}^{T}R_{k}^{-1}(z_{k}-h_{k}(x_{k}^{i^{*}}))}, \end{array}$$

where (**z** − **h**(**x**)) is the difference between recursive estimation and observation. **R**_*k*_ is the estimation value for the real covariance of the particles, which is an important factor for the particle filter. And the estimation accuracy of particle filter will decline when the estimation covariance diverges from the real particles covariance of the particles.

Generally, **R**_*k*_ is set roughly as a fixed constant to generate the weights of the particles. But this method has its limitation, for the particle covariance may vary in iterative process and will be different in individual local filters. Setting **R**_*k*_ as a fixed constant will cause **R**_*k*_ to diverge from the real covariance of the particles.

In this paper, the adaptive calculation method was proposed to adjust **R**_*k*_ by the covariance of the particles in iterative process. Firstly, a fixed constant **R**_*k*_ is used to generate the basic probability of particles. Secondly, the covariance of these particles is used to generate the new value **R**_*k*_′ by (13). The new value **R**_*k*_′ is used to generate the final weights by (12).

(13) $$\begin{array}{c} R_{k}^{\prime }=\sqrt{\overset{N}{\underset{i=1}{\sum }}w_{k}^{i^{*}}{\left(z_{k}-h_{k}\left(x_{k}^{i^{*}}\right)\right)}^{T}\left(z_{k}-h_{k}\left(x_{k}^{i^{*}}\right)\right)} \end{array}$$

(14) $$\begin{array}{c} w_{k}^{{i^{*}}^{\prime }}=\frac{1}{{\left(2\pi \right)}^{n_{v}/2}\sqrt{\det R_{k}^{\prime }}}e^{-(1/2){\left(z_{k}-h_{k}(x_{k}^{i^{*}})\right)}^{T}{\left(R_{k}^{\prime }\right)}^{-1}(z_{k}-h_{k}(x_{k}^{i^{*}}))}. \end{array}$$

In the improved method, **R**_*k*_ is substituted by the estimation covariance **R**_*k*_′, which is calculated in each iteration to improve the robustness of the particle filter. The adaptive adjustment process of estimation covariance receives a better weight for the particles by recalculation step. Since **R**_*k*_′ is calculated in every iteration step, the improved particle filter has a better robustness. Therefore, the improved distributed particle filter systems are better than the original system in accuracy and robustness.

### 3.2. Information Fusion Method

One of the most important advantages of DPF is that by setting different weights to local filters in the master filter, which can reduce the probability of the particle filter with bad performance. From the principle of information sharing scheme, the weights set to individual local filter can be described by

(15) $$\begin{array}{c} \beta_{1}+\beta_{2}+\cdots +\beta_{n}=1, \end{array}$$

where *β*_*n*_ represents the weights allots to each local filter.

In [10], the weights are allotted based on the innovation of each local filter and are calculated in (4). In this method, the innovations (the difference between observation and estimation) were used to evaluate the performance of the local filter; a larger innovation represents a worse quality of the local filter and allotted a lesser weight in master filter. This method can reduce the affection of the observation noise uncertainty, but this method will fail when the estimation results of the local filters are not accurate enough. Therefore, the weight allocation for individual local filters must consider the estimation performance of individual local filters.

The number of effective particles (*N*_eff_) represents the efficiency of the local filter because the value is calculated by the weight of the particles in each local filter, which is indicated in the following:

(16) $$\begin{array}{c} N_{\text{ef}{\text{f}}_{j}}=\frac{1}{\sum_{j=1}^{N}{\left(w_{k}^{j}\right)}^{2}}, \end{array}$$

where *w*_*k*_^*j*^ is the weight of the particles in each filter, *j* is the index of local filters, and *N* is the number of the local filters.

Then the weight, which represents the estimation performance of individual local filter, can be set based on the *N*_eff_ of each local filter and is calculated as

(17) $$\begin{array}{c} \beta_{j}=\frac{N_{\text{ef}{\text{f}}_{j}}}{\sum_{j=1}^{N}\left(N_{\text{ef}{\text{f}}_{j}}\right)}. \end{array}$$

In conclusion, we proposed the improved distributed particle filter given in Algorithm 1 and it is briefly described in it.

### 3.3. Theoretical Convergence

For the proposed Improved DUPF, the convergence of the particle filter with the distributed implementation is an important issue and requires careful investigation, as it is crucial for the successful applications. In this section, the proof of the mean square convergence of the distributed particle filter to the optimal filter was described. First, the convergence results for particle filter were extended to prove that distributed particle filter convergence to the optimal filter in mean square as the number particles go to infinity.

An extensive treatment of the currently existing mean square convergence results of particle filter can be found in [17] and [18] These convergence results demonstrate that, under very loose assumptions, convergence of the particle filter is ensured and that the convergence rate of the method is independent of dimension of the state space. The convergence results for particle filter are described as follows.

Let *B*(*R* ^*n*^) be the space of bounded, Borel measurable functions on *R* ^*n*^ and denote ||*f*||≜sup⁡_*x*∈*R*^*n*^_ | *f*(*x*)|. Then the following theorem is a consequence result of the convergence of particle filter.

**Theorem 1**

If the importance weight

(18) $$\begin{array}{c} w_{t}\propto \frac{p\left(y_{t}\;|\;x_{t}\right)p\left(x_{t}\;|\;x_{t-1}\right)}{q\left(x_{t}\;|\;x_{0:t-1},y_{1:t}\right)} \end{array}$$

is upper bounded for any (**x**_*t*−1_, **y**_*t*_), then for all *t* ≥ 0, there exists *c*_*t*_ independent of *N* such that for any *f*_*t*_ ∈ *B*(*R* ^*n*^)

(19) $$\begin{array}{c} E\left[{\left(\frac{1}{N}\overset{N}{\underset{i=1}{\sum }}f_{t}(x_{0:t}^{i})-\int f_{t}(x_{0:t})p(dx_{0:t}\;|\;y_{1:t})\right)}^{2}\right]\leq c_{t}\frac{{||f_{t}||}^{2}}{N}. \end{array}$$

The convergence of distributed particle filter is as follows: for any fixed time instance *t*, under what conditions and for what kind of function *f*(*x*) does the distributed particle filter approximation converge to the optimal filter? As mentioned above, distributed particle filter is configured by a number of local particle filters. Therefore, the convergence of distributed particle filter can be proved using Theorem 1 and Deduction 1 for the convergence results of particle filter. In the following, the convergence result for distributed particle filter was proved based on Theorem 1.

**Deduction 1**

If all of the local particle filters in distributed particle filter are convergence, the distributed particle filter is surely convergence.

**Proof**

From the mean square convergence, the distributed particle which filter converges to the optimal filter is the estimation results approximate to the conditional expectation *E*(*f*_*t*_(**x**_0:*t*_)/**y**_1:*t*_), which can be calculated by

(20) $$\begin{array}{c} E\left(\frac{f_{t}\left(x_{0:t}\right)}{y_{1:t}}\right)=\int f_{t}\left(x_{0:t}\right)p\left(dx_{0:t}\;|\;y_{1:t}\right). \end{array}$$

From Algorithm 1, the conditional expectation estimated by distributed particle filter $\hat{E}\left(f_{t}\left(x_{0:t}\right)/y_{1:t}\right)$ can be calculated by

(21) $$\begin{array}{c} \hat{E}\left(\frac{f_{t}\left(x_{0:t}\right)}{y_{1:t}}\right)=\frac{1}{N}\overset{N}{\underset{i=1}{\sum }}f_{t}^{q}\left(x_{0:t}^{i}\right)=\frac{1}{N}\overset{m}{\underset{q=1}{\sum }}\beta_{m}\overset{N}{\underset{i=1}{\sum }}f_{t}^{q}\left(x_{0:t}^{i}\right), \end{array}$$

where, *q* = 1,2,…, *m* is the number of the local filter.

Thus, the mean square can be calculated in the following:

(22) E[(E^(ft(x0:t)y1:t)−E(ft(x0:t)y1:t))2] =E[(1N∑q=1mNeffq∑q=1mNeffq ∑i=1Nftq(x0:ti)    −∫ft(x0:t)p(dx0:t ∣ y1:t))2] =E[(1N∑q=1mNeffq∑q=1mNeffq∑i=1Nftq(x0:ti)    −∑q=1mNeffq∑q=1mNeffq∫ftq(x0:t)p(dx0:t ∣ y1:t)    +∑q=1mNeffq∑q=1mNeffq∫ftq(x0:t)p(dx0:t ∣ y1:t)    −∫ft(x0:t)p(dx0:t ∣ y1:t))2],

when *N* → *∞*, there is

(23) ∑q=1mNeffq∑q=1mNeffq∫ftq(x0:t)p(dx0:t ∣ y1:t)  −∫ft(x0:t)p(dx0:t ∣ y1:t)=0.

Thus, we have

(24) E[(1N∑q=1mNeffq∑q=1mNeffq∑i=1Nftq(x0:ti)   −∑q=1mNeffq∑q=1mNeffq∫ftq(x0:t)p(dx0:t ∣ y1:t))2]  =E[(∑q=1mNeffq∑q=1mNeffq     ×(1N∑i=1Nftq(x0:ti)−∫ftq(x0:t)p(dx0:t ∣ y1:t)))2]  =∑q=1mNeffq∑q=1mNeffqE    ×[((1N∑i=1Nftq(x0:ti)−∫ftq(x0:t)p(dx0:t ∣ y1:t)))2],

where, **E**[(((1/*N*)∑_*i*=1_^*N*^ *f*_*t*_^*q*^(**x**_0:*t*_^*i*^) − ∫*f*_*t*_^*q*^(**x**_0:*t*_)*p*(*d* **x**_0:*t*_ | **y**_1:*t*_)))^2^] is the mean square for each local filter. From Theorem 1, the local filters are convergence; we have

(25) E[((1N∑i=1Nftq(x0:ti)−∫ftq(x0:t)p(dx0:t ∣ y1:t)))2]  ≤ct||ftq||2N.

Then, (24) is changed to be

(26) E[(1N∑q=1mNeffq∑q=1mNeffq∑i=1Nftq(x0:ti)−∫ft(x0:t)p(dx0:t ∣ y1:t))2]  ≤∑q=1mNeffq∑q=1mNeffqct||ftq||2N.

It is obvious, from (26), that the distributed particle filter will converge to the optimal filter when all of the local filters are convergence.

## 4. Experiment Results

In this section, the proposed Improved DPF-SLAM system was tested in three experiments. The first experiment was used to test the estimation accuracy by comparing it with DPF-SLAM. The second experiment was used to test the robustness of the proposed Improved DPF-SLAM by adding disturbance noise to the estimation process. The third experiment was used to test the tolerance of the Improved DPF-SLAM by comparing it with the exiting methods.

**Figure 3:**
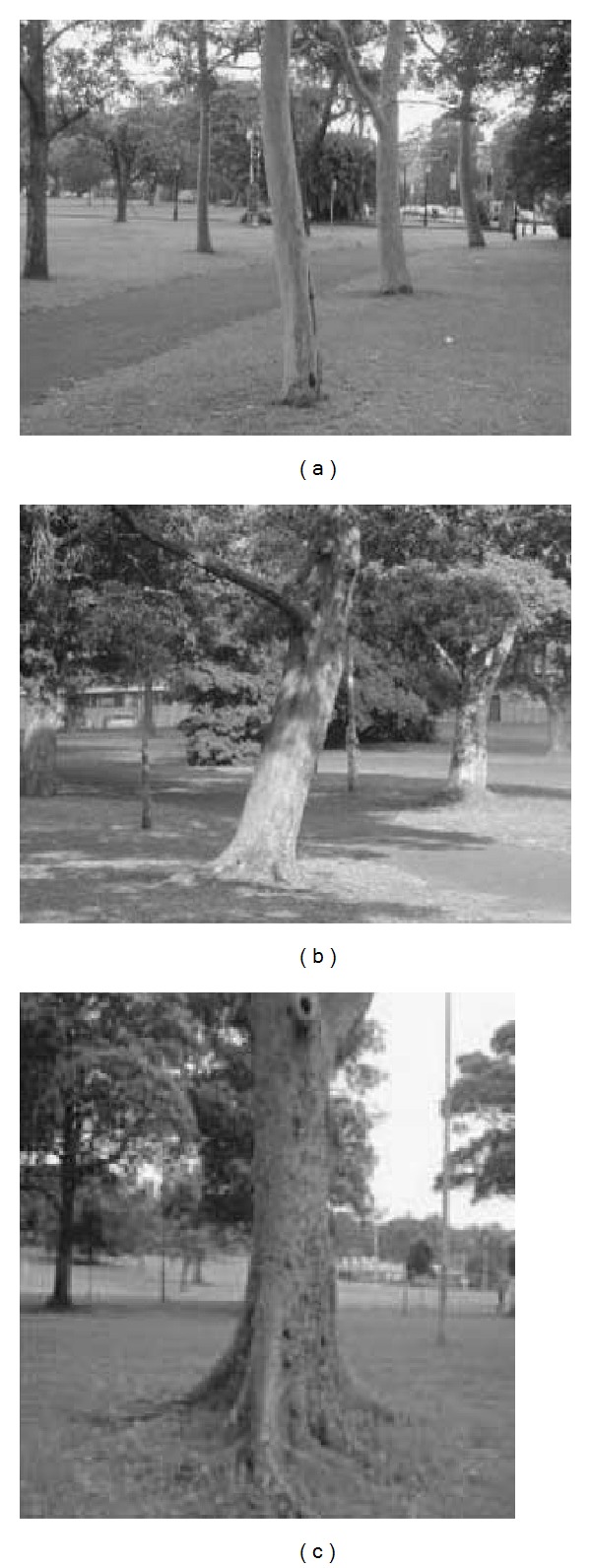
Trees with different shape, size and inclination.

**Figure 4:**
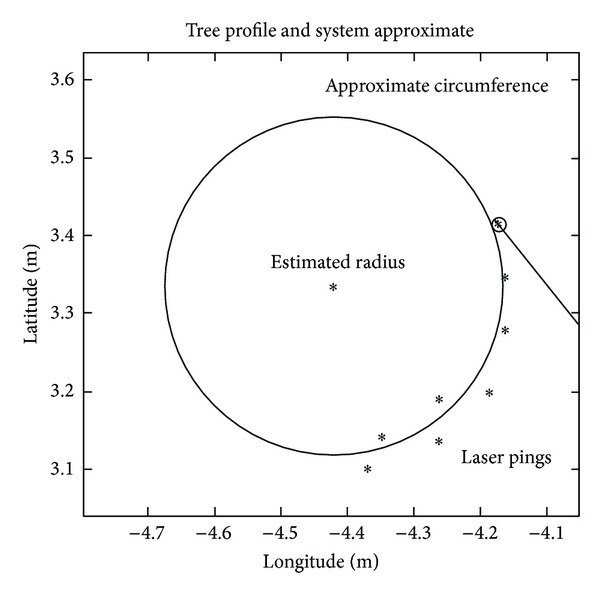
Tree information and trunk approximation.

These experiments were tested using the experiment data that come from an experiment that was finished in Victoria Park in Sydney, Australia [19, 20]. The trees can be considered one of the most relevant features that a laser range sensor can identify in this outdoor environment. The algorithm implemented in [17] was used in this paper, which tracks the center of the trunk by clustering a number of laser observations. The extract algorithm was shown in Figures 3 and 4.

**Experiment 1**

To test the performance of the proposed improved DPF-SLAM, the experiment was finished to be compared with DPF-SLAM.

**Figure 5:**
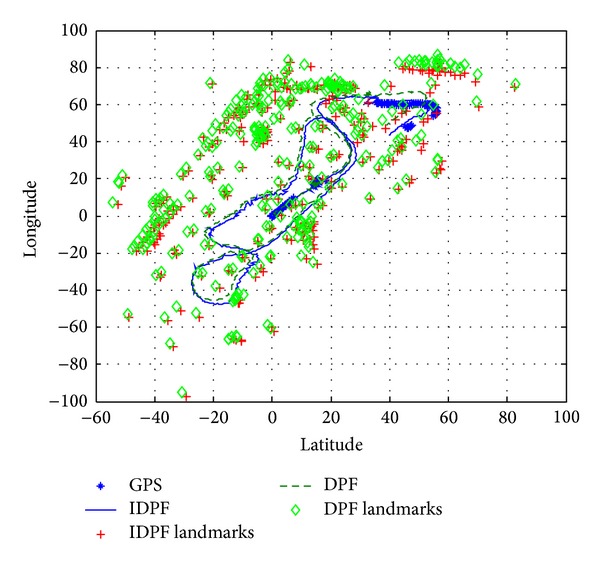
The comparison of estimation results in Experiment 1.

Figure 5 describes the experiment results of Improved DPF-SLAM and DPF-SLAM. In Figure 5, the straight line indicates the estimation results of robot's position from Improved DPF, and the plus sign indicates the estimation results of feature points' position from Improved DPF. Correspondingly, the dashed line indicates the estimation results of robot's position from DPF, and the rhombus indicates the estimation results of feature points' position from DPF. The stars indicate the measurement results from GPS, which are used as the reference information for these experiments.

From Figure 5, it is obvious that the estimation results of Improved DPF-SLAM are more approximate to the results of GPS than DPF-SLAM, which means that the Improved DPF-SLAM has better accuracy than DPF-SLAM.

Table 1 describes the mean value and variance of the estimation results from these two algorithms. From Table 1, it is obvious that the estimation results from Improved DPF are smaller than the estimation results from DPF in mean and variance, which also means that the Improved DPF-SLAM has better accuracy than DPF-SLAM.

**Experiment 2**

To test the robustness of the Improved DPF-SLAM, the experiment was finished and the experiment data used in Experiment 2 were the same as Experiment 1. The disturbance noise was added into the SLAM system process from the 1000th step.

**Figure 6:**
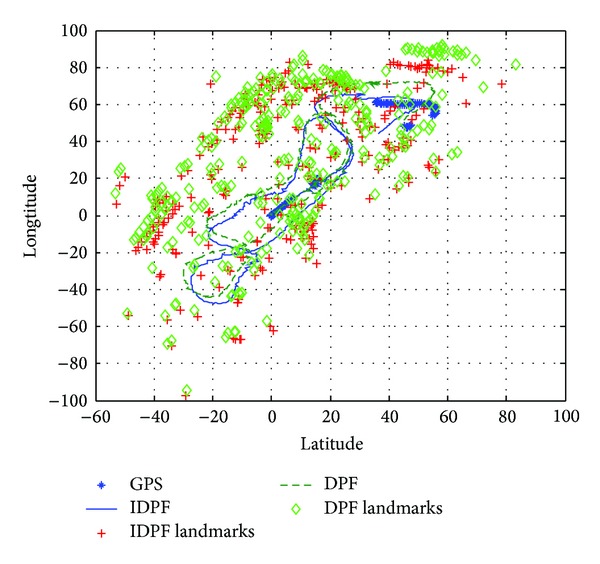
The comparison of estimation results in Experiment 2.

Figure 6 describes the estimation results using Improved DPF-SLAM and DPF-SLAM. In Figure 6, the straight line indicates the estimation results of robot's position from Improved DPF, and the plus sign indicates the estimation results of feature points' position from Improved DPF. Correspondingly, the dashed line indicates the estimation results of robot's position from DPF, and the rhombus indicate the estimation results of feature points' position from DPF. The stars indicate the measurement results from GPS, which are used the reference information for these experiments.

From Figure 6, it is obvious that the estimation results of Improved DPF-SLAM are more approximate to the results from GPS than DPF-SLAM, which means that the Improved DPF-SLAM has better robustness than DPF-SLAM in disturbance condition.

Table 2 describes the mean value and variance of the estimation results from these two algorithms. From Table 2, the estimation results from Improved DPF are obviously smaller than the estimation results from DPF in mean and variance, which also means that the Improved DPF-SLAM has better performance than DPF-SLAM in disturbance condition.

**Experiment 3**

To test the tolerance of the Improved DPF-SLAM, the experiment was finished and the experiment data used in Experiment 2 were the same as Experiment 1. The disturbance noise was added into the SLAM system process from the 1000th step.

**Figure 7:**
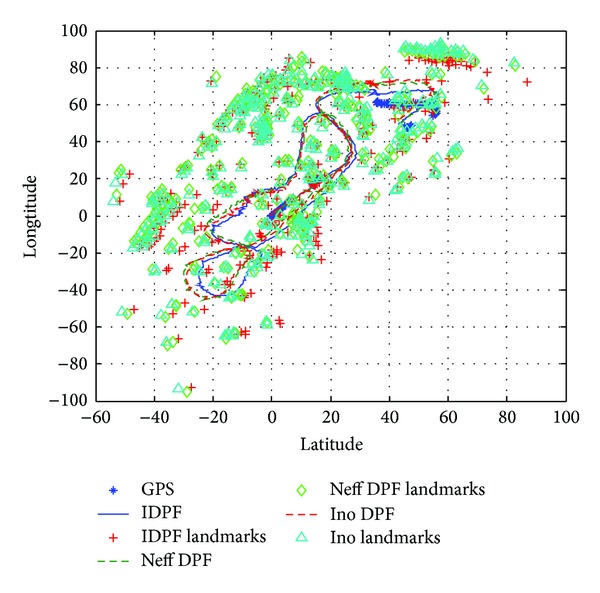
The comparison of estimation results in Experiment 3.

Figure 7 describes the estimation results using Improved DPF-SLAM and DPF-SLAM. In Figure 7, the straight line indicates the estimation results of robot's position from Improved DPF, and the plus sign indicates the estimation results of feature points' position from Improved DPF. Correspondingly, the dashed line indicates the estimation results of robot's position from DPF using *N*_eff_ method, and the rhombus indicates the estimation results of feature points' position from DPF using *N*_eff_ method. And the dotted line indicates the estimation results of robot's position from DPF using Innovation method, and the triangles indicate the estimation results of feature points' position from DPF using Innovation method. The stars indicate the measurement results from GPS, which are used as the reference information for these experiments.

From Figure 7, it is obvious that the estimation results of Improved DPF-SLAM are more approximate to the results from GPS than DPF-SLAM using *N*_eff_ method or using Innovation method, which means that the Improved DPF-SLAM has better robustness than these two methods.

Table 3 describes the mean value and variance of the estimation results from these three algorithms. From Table 3, it is obvious that the estimation results from Improved DPF are smaller than the estimation results from other methods in mean and variance, which also means that the Improved DPF-SLAM has better accuracy than other methods.

## 5. Conclusion

In this paper, we proposed an improved particle filter for distributed SLAM to generate a map and localize the robot. Firstly, the variance estimation of particle distribution in local particle filters is a vital parameter in generating the weights of the particles. An optimized variance estimation of particle distribution will ensure that the particle filter has a better performance. Instead of a fixed parameter in variance estimation, the particles variance was adjusted with the variance estimation adaptively. Secondly, the weight of local filters in master filter is also an important factor in distributed particle filter. The information fusion method mixing the *N*_eff_ method and the innovation method were proposed by this paper. To test the performance of the Improved DPF-SLAM algorithm, three experiments were designed. The experiment results show that the improved DPF-SLAM algorithm has the advantage in robustness and accuracy and has the better tolerance than the DPF-SLAM algorithm.

## Figures and Tables

**Algorithm 1:**
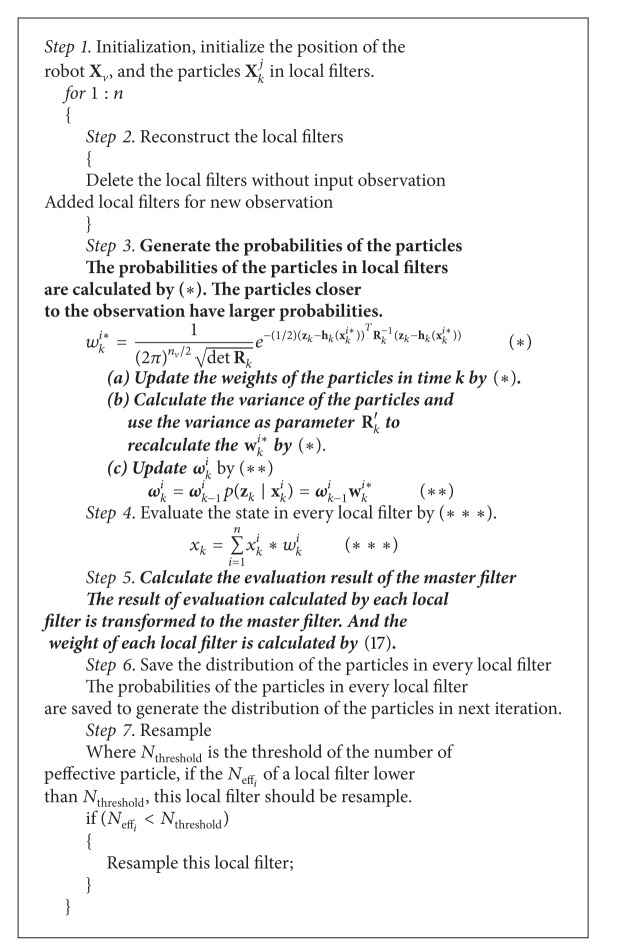
Improved distributed particle filter (Improved DPF).

**Table 1:** The comparison of mean and variance in Experiment 1.

Algorithm	MSE	
	Mean	Variance
Improved DPF	2.2367	2.2367
DPF	3.65898	3.65898

**Table 2:** The comparison of mean and variance in Experiment 2.

Algorithm	MSE	
	Mean	Variance
Improved DPF	3.92396	3.18096
DPF	5.41226	4.7737

**Table 3:** The comparison of mean and variance in Experiment 3.

Algorithm	MSE	
	Mean	Variance
Improved DPF	3.3139	3.1364
*N*_eff_ DPF	4.65898	4.75815
Ino DPF	3.31508	3.20737
